# Monthly engagement with EIP keyworkers was associated with a five-fold increase in the odds of engagement with psychosocial interventions

**DOI:** 10.1186/s12888-024-05577-7

**Published:** 2024-02-05

**Authors:** C. D. Darker, G. Nicolson, H. Reddon, K. O’Connor, R. Jennings, N. O’Connell

**Affiliations:** 1 Discipline of Public Health and Primary Care, Institute of Population Health, School of Medicine, Trinity College Dublin, Dublin, Ireland; 2 Health Promotion and Improvement Department, HSE Health and Wellbeing, 1st Floor Old National Ambulance Training Building, St Marys Hospital Campus, Phoenix Park, Dublin 20, Chapelizod, D20 TY72 Ireland; 3 Department of Medicine, University of British Columbia, Vancouver, Canada; 4 National Clinical Programme for Early Intervention in Psychosis, Health Service Executive Dublin, Dublin, Ireland; 5 Rise, South Lee Mental Health Services, Cork & Department of Psychiatry, University College Cork, Cork, Ireland; 6 Sexual Health and Crisis Pregnancy Programme, HSE Health and Wellbeing, Strategy and Research, 89-94 Capel St, Dublin 1, Dublin, D01 P281 Ireland

**Keywords:** Early intervention in psychosis, First episode psychosis, Keyworker, Care coordinator, Model of care, Engagement, Qualitative, Process evaluation, Demonstration sites

## Abstract

**Background:**

Early intervention in psychosis (EIP) supports people who are experiencing their first episode of psychosis (FEP). A new Model of Care (MoC) for EIP services was launched in Ireland in 2019. Three EIP demonstration sites were chosen to test this MoC through a ‘hub and spoke’ approach. These services were a new way of organising care for people experiencing FEP, based upon a recovery model of care, and which sought to standardise care, improve access by clinically led multidisciplinary teams. This included newly created EIP keyworker roles whereby keyworkers assumed responsibilities regarding assessment, comprehensive individual care planning and coordination of care.

**Methods:**

A mixed methods design utilising the UK Medical Research Council’s process evaluation framework. Purposive sampling techniques were utilised. Descriptive analyses and logistic regression were performed to examine how increased keyworker engagement influenced the use of other psychosocial interventions within the EIP demonstration sites. Thematic analyses was used for qualitative data.

**Results:**

There was a strong positive relationship between keyworker contacts and psychosocial interventions offered. Specifically, the odds of achieving at least monthly engagement with cognitive behavioural therapy for psychosis (CBTp; (5.76 (2.43–13.64), *p* < 0.001), and behavioural family therapy (BFT; (5.52(1.63–18.69, *p* < 0.006)) increased by fivefold with each additional monthly keyworker contact. For individual placement support (IPS) each additional monthly keyworker contact was associated with a three-fold increase in the odds of achieving monthly attendance with IPS (3.73 (1.64–8.48), *p* < 0.002). Qualitative results found that the EIP keyworker role as viewed by both service users and staff as a valuable nodal point, with a particular emphasis on care coordination and effective communication.

**Conclusions:**

This study advances the understanding of keyworker effects through qualitative evidence of keyworkers functioning as a “linchpin” to the service, while the positive response association between keyworker contacts and engagement with other services provides quantitative support for keyworkers reducing the organisational or structural barriers to service access. Given the importance of these positions, health systems should ensure that EIP programmes identify qualified and experienced staff to fill these roles, as well as allocate the appropriate funding and protected time to support keyworker engagement and impact.

## Background

Early intervention in psychosis (EIP) services prioritises detection, quick assessments, and reduced waiting times to facilitate timely access to high-quality care for people experiencing their first episode of psychosis (FEP; [1]). EIP services were first introduced in the United Kingdom in the early 2000s [2], in Canada from 2004 [3] and in Australia as early as the 1980s [4]. Ireland’s first EIP service was developed in 2005 but a lack of committed funding and resources impeded their full implementation [5]. A Model of Care (MoC) was developed and published in 2019 which committed to developing the standards of care, for those aged 18–64 years old, shared across EIP services, i.e., to optimise care for all service users experiencing FEP; to improve detection rates and reduce delays in accessing treatment; to lower risks of progression to more enduring states of psychosis; improve rates of remission; reduce rates of hospitalisation; improve satisfaction with the service and reduce physical complications [6].

A recent meta-analysis of EIP services compared with treatment as usual found that EIP services were associated with better outcomes including all-cause treatment discontinuation, improved rates of involvement in school or work, total symptom severity, and reduced hospital admissions [7]. In Ireland, EIP services can be provided through standalone teams or through a ‘hub and spoke’ MoC. Given Ireland’s population is widely dispersed and often rural, with areas of very low population density, a hub and spoke model was deemed to be most appropriate for delivering EIP services in non-urban areas. It has been noted, however, that there can be challenges in engaging people located far away from standalone centres which can require long distance [8]. In the hub and spoke model, EIP services are located within the hub under the clinical leadership of a consultant psychiatrist and supported by EIP roles such as a keyworker, behavioural family therapy (BFT) and a clinician delivering cognitive behavioural therapy with a focus on psychosis (CBTp) and individual placement support (IPS) – all of whom may be located in the hub and /or spokes. The pillar interventions of EIP have equal weighting and include medication, psychosis-specific psychological interventions, psychosis-specific family support and interventions, physical health screening and intervention, and employment support.

The spokes comprise the existing community mental health teams (CMHTs) and their multidisciplinary specialisms which feed into and support the hub. EIP staff meet in their specialty hubs to discuss cases, receive training and supervision and conduct meetings, but their routine day-to-day EIP work with service users is carried out across the hub and the spokes.

Given the multidisciplinary nature of EIP services, the care coordinator or keyworker plays an important role in the effective delivery of EIP care [9]. The keyworker is an experienced senior mental health professional with a least 3 years experience working in community mental health settings. Keyworkers were recruited from the disciplines of mental health nursing, social work or occupational therapy. It is a non-discipline specific clinical role that supports the service user and their family through a single point of contact. Caseloads are set at about 15 cases per keyworker. This is to allow for intense engagement with the service user and their carer/family/support person, and to ensure uptake of evidence based interventions on offer at EIP services – CBTp, BFT, IPS, physical health monitoring and medication.

Keyworkers play a central role in engagement, comprehensive clinical assessment and ensuring individual care planning, integration across services and continuity across services. As many people who experience FEP have complex needs, a dedicated EIP keyworker is crucial to avoid potential risks of care fragmentation in people with complex needs. Service users may need diverse treatments and interventions at different times or stages within their recovery to best support them across the total spectrum of their needs. Key workers play other facilitative roles such as helping to reduce comorbid substance misuse, and enhancing engagement in discussions on physical health [10]. Better therapeutic relationships are also associated with better adherence to antipsychotic medications [11]. Unlike in other jurisdictions, keyworking is not routine in the Irish mental health service. Therefore, in the current study, clinicians appointed were testing this new role outside of their core training. All EIP keyworkers had access to an EIP induction training programme which included e-modules on topics such as EIP case management, case formulation, crisis intervention and risk management, plus working with families. Quarterly webinars were delivered on topics such as dual diagnosis, psychological interventions, trauma and psychosis etc. EIP keyworkers also had access to monthly supervision delivered online by an external facilitator.

A descriptive review of thirty-one qualitative papers found that for people experiencing FEP it can be a complex series of social and psychological processes that individuals experience including achieving and incorporating psychosis into their identity, acquiring new perspectives on what psychosis is, and developing relationships with family and friends during this significant time period of their lives [12]. Qualitative studies of EIP services have reported a number of factors that service users value about EIP. Harris et al. found that service users wished for services to facilitate opportunities for them to have more agency and control over their treatment plans and recovery [13]. Lester et al. interviewed young service users early in their engagement with services and then again a year later, reporting that service users valued the relationships with key workers, as well involvement and support from their family [14]. A recent paper called for an exploration of family members’ and staffs’ thoughts on the components of treatment viewed as most helpful and important [15].

The establishment of a new MoC within any existing public health service represents a complex intervention. A process evaluation of complex interventions provides information on how interventions work both together and as standalone programmes, and the conditions which shape implementation of the intervention and future outcomes. Process information on such interventions is critical to decision makers and service providers who may seek to later embed the MoC nationally within usual care settings. This study presents findings from a process evaluation of three demonstration EIP services in Ireland.

The aim of this paper was to explore and understand from service users’ and other clinical team members’ perspectives, their views and experiences of the EIP keyworker role in the context of a newly established EIP service and to examine the effect the keyworker role has on engagement of service users with other EIP services such as CBTp, BFT and IPS.

## Methods

### Design

The design of this study was informed by the UK Medical Research Council’s process evaluation guidelines for the evaluation of complex interventions [16]. The overall evaluation utilised three methodologies, which included, desk based review of existing EIP documentation within the service; quantitative data from demonstration sites and qualitative interviews [17]. In terms of the qualitative method, stakeholder interviews are a valuable way for process evaluation inquiry to capture the experiences and unanticipated or complex emerging changes and mechanisms in implementation [16]. Quantitative methods were used to measure key process variables and to assess mechanisms of impact and contextual moderators.

### Setting

In January 2018, three demonstration sites were selected from an open application process by the Health Service Executive’s (HSE) National Clinical Programme to test the ‘hub and spoke’ model in practice, with limited additional resources for each site selected. Each demonstration site included both a hub and numerous spokes. To protect the anonymity of the research sites, they will be referred to as Site A, B, and C. Site A was more established and tends to see a more urban and larger population size than Site B.

### Procedure

A data capture database was designed to facilitate clinical staff to record routinely collected data such as referral sources, time to first assessment etc. Ireland does not have electronic health records across much of its health service including community care; with patient data routinely kept in paper records and individual practitioner case files [18–20]. This database captured service user level information such as socio-demographic data as well as monthly service level activities such as service users’ engagement with keyworkers. An individual ID was created for each service user to help track their care pathway on a monthly basis through the new services. Team members were encouraged to enter data on a monthly basis. All team members (peer support, psychological services, BFT and IPS) could input their activity into the database to capture service users’ engagement with each programme. Data entry for all service users presenting to Site A started in June 2020, and in Site B in January 2021. Data entry stopped in both sites in December 2021. A total of 192 service users’ information was captured during this time period. Monthly aggregated data assessed trends in referrals (e.g., the number of EIP referrals each month, the type of referrers), the number of monthly assessments that took place, the time between receipt of referral and the assessment, the number of ‘Did not attends’ (DNAs), the interventions offered by the EIP team and the number of interventions attended, as well as a range of information on the service user themselves (e.g., their receipt of benefits, their diagnostic status, their inpatient and adverse event history and their discharge status). Information relating to the engagement of service users with different treatment options available (e.g., keyworker contacts; engagement with CBTp etc) was considered an important factor in understanding the effectiveness of the programme.

Participants who were service providers were invited to participate in the qualitative component of the study through an email sent by the research team. The service users’ keyworker invited the service users and their family members to participate in the study at an appropriate time in the service users’ care pathway. A semi-structured interview schedule guided the interviews. The interview questions were derived via consultation with the National Clinical Lead, a thorough review of the literature, and discussions within the research team. All of the interviews were conducted and recorded via Zoom between February 2020 and February 2022. The recordings were professionally transcribed. Prior to commencing the interviews, informed written consent was obtained. The issue of confidentiality is key when conducting interviews from a limited pool of participants, therefore care was taken to anonymise any identifiable information. Names were changed and participants were referred to by their role, e.g. CBTp1 and the Site from which they were located.

### Participants

In relation to the quantitative data, a total of 192 service users’ information was collected (Site A *n* = 141, Site B *n* = 51). Each service users’ clinical pathway began to emerge as their contact with each keyworker and clinician was captured on a monthly basis. No prevalence data existed for FEP in Ireland during the planning stages of the project. Therefore, no a priori sample size for the quantitative data was set at the start of the study.

The sampling procedure for the qualitative component targeted key stakeholders involved with EIP. A total of 40 participants were interviewed across Site A, B and C, which included 22 EIP service providers, and nine management and administrative representatives. Eight service users and one family member of a service user participated in the study. Purposive sampling was employed to gain insight and experience from a variety of knowledgeable individuals involved with and engaging in the service, to provide detailed understandings of the functioning of the intervention.

### Analyses

Descriptive analyses and a number of exploratory analyses were performed to summarise and make sense of the data as it emerged. Analysis of key individual level socio-demographic data and service level data relevant to the MoC, such as, trends in referrals (e.g., the number of EIP referrals each month, the type of referrers) and assessment outcomes were completed. Caseload data, the interventions offered by the EIP team, information relating to the engagement of service users with different treatment options available (e.g., keyworker contacts; engagement with CBTp etc.) to understand factors within the programme were explored.

To evaluate the impact of keyworker engagement among EIP service users, logistic regression analyses were conducted to examine how increased keyworker engagement influenced the use of cognitive behavioural therapy for psychosis (CBTp), behavioural family therapy (BFT) and individual placement and support services (IPS). The primary explanatory variable was the average number of keyworker contacts per month that took place per service user during 2021. Separate analyses were conducted with CBTp, BFT and IPS as the dependent variable. These variables were measured as the average number of contacts with each of these services per month that took place during 2021. For the purposes of these analyses, these variables were dichotomized with values of “1” representing service users who averaged at least monthly contact with these services. These analyses were adjusted for sex, age, duration of untreated psychosis, housing status (living alone vs. living with family or partner), employment (employed/in education vs. unemployed) and substance use in the last four weeks (yes vs. no). All statistical analyses were performed using SPSS version 21 (IBM Corporation, New York, USA). All tests of significance were two-sided with a significance threshold of *p* < 0.05.

Thematic analyses was used as the qualitative analytical method. This method involves careful engagement with interview transcripts and recordings to identify patterns in meaning across the data to derive themes [21]. It is a very flexible approach that enables researchers to generate new insights and concepts derived from data. The six steps of thematic derision was followed which includes familiarisation with the data; generating initial codes; searching for themes of a broader significance; reviewing themes to combine, modify, divide or even discard; defining and naming theme narratively; and writing up the final analyses and description of the findings [21]. NVIVO software (QSR International Pty Ltd. Version 12, 2018) was used to manage and organise the data to facilitate the development of a robust coding framework.

### Research ethics

The overall study was approved by the Royal College of Physicians of Ireland (reference RCPI RECSAF 79). Research ethical approval was also required from local research ethics committees associated with the three demonstration sites for the quantitative data collection procedures. Two of the three research ethics committees deemed the quantitative data collection as being ‘low risk’ and therefore not requiring individualised consent and approved those protocols (Clinical Research Ethics Committee of Site A Teaching Hospitals, and Research Ethics Committee, Site B University Hospital). However, the research ethics committee associated with the third demonstration site (HSE Site C Research Ethics Committee) deemed the quantitative data collection as ‘high risk’ requiring individualised consent from each service user for their data to be used in the study. We therefore only present quantitative data from two of the three demonstration sites.

## Results

### Quantitative description of service users, case load service, engagement, and response data

#### Socio-demographic description of service users

The mean age of service users was 35.1 years (sd 12.5 years) in Site A and 32.0 years (sd 12.1) in Site B (see Table 1). The majority of the participants were male (Site A: 63%, Site B: 56%), single (Site A: 73%, Site B: 89%) and reported living with family or a partner (Site A: 63%, Site B: 56%). The proportion of service users who were medical card holders (i.e., citizens who are entitled to access primary and secondary care services for free and for low cost prescriptions) was 30% in Site A and 21% in Site B. Rates of employment and engagement in education were also greater in Site A (employed: 31%, education: 12%) compared to Site B (employed: 21%, education: 4%).

**Table 1:** Socio-demographic description of service users, and referral and assessment outcomes in two EIP demonstration sites (*N* = 192)

Variable	Site A (*N* = 141) (pop.est.200,000)	Site B (*N* = 52) (pop.est.115,000)
**Demographic data**		
Age in years (SD)	35.1(12.5)	32.0(12.1)
Male (%)	89(63)	29(56)
Marital status – single (%)	103(73)	46(89)
Living with family/partner (%)	103(72)	41(79)
*Medical card holders (%)	42(30)	11(21)
Employed	44(31)	11(21)
Engaged in education	17(12)	2(4)
**Referral and assessment outcomes**		
Community mental health team referrals (%)	25(18)	38(74)
EIP assessment completed ≤ 3 days (%)	80(57)	18(34)
Duration of untreated psychosis		
< 1 month < 6 months < 1 year > 1 year	45 31 13 29	8 5 5 17
Past month substance use (%)	56(40)	19(36)
Illicit drug use (%)	27(19)	7(14)
Alcohol use alone (%)	11(8)	4(7)
Most common antipsychotic prescribed – Olanzapine (%)	30(21)	20(38)
Any antipsychotic	86(61)	39(75)

*citizens who are entitled to access primary care and secondary care services for free, with low cost prescriptions

#### Referral and assessment outcomes

Approved care centres provided the most referrals for Site A compared to CMHTs for Site B. At Site A, 57% of service users had their initial assessments completed within three days, whereas only 34% met this criteria at Site B. The majority of service users in Site A had experienced untreated psychosis for less than one months, whereas the majority of service users in Site B had experienced untreated psychosis for more than a year. Past month substance use was reported by 40% of service users at Site A and 36% of service users at Site B, with 19% and 14% of participants reporting illicit substance use at Sites A and B, respectively. Olanzapine was the most common antipsychotic medication prescribed at both sites (Site A: 21%, Site B: 38%) and the proportion of service users receiving any antipsychotic medication was 61% at Site A and 75% at Site B.

#### Case load

Since there was not a focus on EIP or FEP under treatment as usual (TAU) conditions, it was not possible establish caseloads prior to the implementation of the MoC. This was attributed to the lack of keyworkers and keyworking duties being performed under TAU condition in Ireland. Therefore, caseloads related to these activities were not recorded prior to testing the EIP services within the demonstration sites.

The caseloads for keyworkers were approximately 20 in Site A (the MoC guideline is 15 service users per keyworker), 10–12 in Site B and 5–14 in Site C, where there were four keyworkers in post. During 2021 in Site B, there was a period where new service users could be accepted into interventions but could not be assigned a keyworker due to problems with recruiting staff into posts and releasing funds to support the budget of the service. There was also a freeze on accepting new service users in Site A during October of 2021 due to limited capacity caused by delays in hiring processes and an unfilled maternity leave. Caseloads were frozen on the basis of capacity and risk. Waiting lists also developed in Site C due to delays in the identification, recruitment and agreed start dates of staff in post for the EIP team. It is also important to acknowledge that caseloads were highly variable during the process evaluation and the qualitative data indicated that this variability was mainly due to barriers with recruitment and budgeting challenges (paper in preparation).

#### Service level contact with keyworkers, and psychosocial interventions

The mean number of EIP keyworker contacts completed by the service users was five per month (range of 0–12). Telephone contact (at least 15 min) was the most common method of contact with keyworkers in 2021. Site A received an average of 1.4 calls per month while Site B averaged 1.9 calls per month. Overall, participants attended 79% of the psychological appointments offered to them. Due to the COVID-19 pandemic and Government public health guidelines, in-person visits were limited and many service users shifted to telephone and virtual communication with keyworkers. There were also three keyworkers who were on maternity leave at Site A for periods of 2021 and this created a barrier to keyworker contacts for some of the service users.

The average proportion of service users engaged with monthly psychological interventions ranged from 78% in Site A to 56% in Site B. Psychological interventions were declined by few participants (Site A = 3.7%, Site B = 5.5%) and a small proportion of service users completed their psychological interventions during 2021 (Site A = 8.3%, Site B = 2.7%).

There were significant fluctuations in BFT engagement during the implementation of the model of care. The qualitative data demonstrated that the service delivery interruptions caused by COVID-19 public health measures likely accounted for this variation. There was a high acceptance of BFT among service users, with an average of 60% (sd = 20) attending BFT among those who were offered these sessions across both sites. BFT engagement was defined as participation in at least one session per month and the average number of service users engaged in BFT was 18 (sd = 5) in Site A and 6 (sd = 3) in Site B.

Similar to BFT, IPS engagement was defined as participation in at least one IPS session per month. Throughout 2021, IPS engagement generally increased with an average 20 (sd = 4) service users engaged at Site A and 12 (sd = 3) in Site B. Of the service users engaged in IPS, an average of 49% (sd = 25) were able to secure employment.

Association between keyworker engagement and psychosocial interventions offered.

We observed a strong positive association between the number of monthly keyworker contacts with service users and the number of psychosocial interventions offered, which was independent of demographic factors which were controlled for in the analyses. Each additional monthly keyworker contact was associated with a five-fold increase in the odds of achieving at least monthly engagement with CBTp (OR = 5.76, 95% CI: 2.43–13.64, *p* < 0.001) and BFT (OR = 5.52, 95% CI: 1.63–18.69, *p* < 0.006). With respect to IPS, we observed a three-fold increase in the odds of achieving monthly attendance associated with each additional monthly keyworker contact (OR = 3.73, 95% CI: 1.64–8.48, *p* < 0.002) (see Table 2).

**Table 2:** Logistic regression analysis of the association between number of monthly keyworker contacts and engagement with CBTp, BFT and IPS.

	Adjusted	
Outcome variable	**Odds Ratio** **(95% CI**)	***p -*** **value**
CBTp	5.76 (2.43–13.64)	**< 0.001**
**BFT**	5.52 (1.63–18.69)	**0.006**
IPS	3.73 (1.64–8.48)	**0.002**

Notes: CBTp = cognitive behavioural therapy for psychosis, BFT = behavioural family therapy, IPS = individual placement and support, CI = confidence interval. All analyses were adjusted for sex, age, duration of untreated psychosis, housing status, employment and substance use in the last four weeks

### Qualitative data

#### Care coordination and communication

Experiences of EIP staff in relation to their perceptions of the role of the keyworker were largely positive. All respondents recognised the significance of key working and identified the need to have a clear job description underpinning the role.

*“The keyworker is central to the whole thing. The keyworker coordinates the medical and the psychosocial aspects of the service users care. They are the linchpin to the whole operation”.* [Site A, Clinician]

There was recognition from staff members about the comprehensiveness in communication and the benefits of having a single member of staff who works intensively with the service user. The keyworker was seen as the nodal point within the team.

*“The main benefit is having someone from the team who knows the service users inside and out. We all get to meet with the service users in our individual areas of practice, but with so many people involved in their care, and even trying to liaise with the families too, there really has to be a coordinator for all of that. [xxxx] is the person that links us all together. Its about the quality of the care that the role can bring. Its really brilliant.”* [Site B, CBTp].

The keyworkers activities were perceived by other team members as being linked to planned tasks, in terms of providing a single point of contact for the service user into the service but likewise team members also came to rely on keyworkers to provide overviews of the progress of the service user in their recovery journey and their continued motivation to engage with the service as a whole.

*“I find that during the team meetings where we are discussing cases we often refer and somewhat defer to the keyworkers to provide some ‘real time’ information in relation to where a particular service user is at, how they are really doing and whether they are ready to start maybe another phase of treatment”* [Site C, BFT].

Service users were also very complementary about the role of the keyworker, particularly citing the level of engagement, the flexibility ways to establish and maintain contact, and seeing them as a link to other team members.

*“[My keyworker] is great, to be honest, I think that she has been really key. She chats back to me, I’ve gone to see people before about my depression and they never really talked back to me, I just got pills. Now, I’ve loads of people to talk too. I talk and she sort of talks back to me and goes through it all with me. She explains it all to me and my family too. I think she genuinely cares whether I do well or not, which is nice. She calls me on the phone a lot, and its very positive, it picks me up when I get a call from her. We’ve even gone for a walk, I’d say that’s because of COVID but it was a nice break from the norm. She links me in with others on the team and talks to me about what those sessions will be like before I have them. Its just amazing really”* [Site B, service user].

#### A defined scope and role

The MoC sets out that a keyworker should have a case load of no more that 15 cases at any one time to facilitate them working closely and comprehensively with each service user.

*“I would say that working as a keyworker is a complicated role. All of the processes that take place from us receiving that referral, arranging the assessment and then commencing treatment. They have a caseload that’s not meant to go over, I think, 15 cases. We have gone beyond that here at a couple of points and you could notice the difference immediately”.* [Site A, Clinician]

Keyworkers can be drawn from a range of different clinical disciplines such as nursing, social work and occupational therapy. The capacity to have keyworkers coming from general disciplines already embedded within the mental health system was seen as a strength.

*“I think that its good that the keyworkers can come from anywhere and role within the service. That hopefully will mean that we can always have a good supply of them. What is a concern is whether the role description is written too broadly and whether we might need to go back and look at revising and refining that again”* [Site C, Clinician].

## Discussion

We observed that the EIP keyworker was a nodal point in coordinating and facilitating engagement with the EIP service. The keyworker role was accepted and valued by other team members and service users generally, with a particular emphasis placed on the dual activities of care coordination and effective communication. Appointments with keyworkers were kept in local community venues, service users’ homes, even local parks rather than in secondary-care hospital settings. COVID-19 had an immediate impact on the provision of EIP services internationally, with services shifting the modality of contact with service users from predominately face-to-face contact to telehealth modes of delivery [22]. Some aspects of service level contacts within the demonstration sites were completed remotely, but service users responded well to this and could recognise the importance of continuity and flexibility by the service to continue treatment during the pandemic. The availability of the keyworker in particular, as an ongoing contact to monitor and coordinate care was commonly referenced as a facilitator of engagement by service users, which is echoed in other research of young people experiencing their FEP [14]. Other research has focused on the perceptions of the keyworker role from the perspective of service users [14], and families. The current paper is only one of two look at the perceptions of the keyworker role from the perspectives of other clinical team members [23].

Keyworker engagement was a significant facilitator of engagement with other professionals within the EIP team. Specifically, monthly engagement with keyworkers was associated with a five-fold increase in the odds of engagement with CBTp and BFT, and each additional contact with keyworker increased the odds engaging with IPS services by three-fold. This evidence is important as it extends recent findings which found that ‘partnerships’ with service users are an essential factor in promoting service-user engagement [24]. It is possible the causal mechanism here is the therapeutic relationship, whereby the stronger the relationship, the more likely a service user is to engage with all aspects of the programme. Previous research has found the therapeutic relationship predicts outcomes of complex psychiatric treatment programmes in service users with psychosis, such as reducing hospitalisation, symptom levels and improving functioning [25]. Thus, the therapeutic relationships is central to psychiatric care. It is the mechanism through which diagnoses are made, treatment plans negotiated and interventions delivered and this research suggests it plays some role in service users accessing all aspects an EIP service has to offer.

The keyworker role is demanding since they are a single point of contact for the service user to help them coordinate their care. This requires time to meet with service users on a regular basis, designing care plans in conjunction with the service user and the EIP team, coordinating the delivery of the plan, responding to service users queries and questions to help them navigate the service, and at times to be present at various meetings and appointments if required.

In terms of engagement, keyworkers achieved an average of five contacts with service users per month; majority of service users were engaged with CBTp (78% in Site A; 56% in Site B), engagement with BFT was well-received (with an average of 60% of those offered BFT availed of the service) and among those who were engaged with IPS, 49% were able to secure employment. While few studies quantify the number of EIP service users who opt-in or engage in CBTp, an average of 16% with drop out over the course of the therapy [26]. Studies have shown that younger service users benefit more from CBTp in terms of positive symptoms [27]and that women benefit more than men in overall psychopathology [28]. It is important to note the possibility of a confounder in our results as those engaged in an EIP service are more likely to engage in its ancillary programmes than those not engaged in EIP services at all. Engagement in EIP services is therefore likely to be a proxy for engagement in all programmes. The higher rates of engagement between demonstration sites for CBTp is of interest however. This study was undertaken during COVID-19, when Ireland like many other countries experienced a series of ‘lockdowns’. In the EIP teams under study, face to face clinical review continued but was supplemented by increased telephone and video call reviews. There was a rapid upscaling in these EIP teams of access to digital resources e.g. laptops, videoconferencing software etc. The decisions to deliver EIP care in person, via phone/ via teleconferencing were influenced by service user preferences, service user access to technology, and clinical risk assessment. Although virtual contact with patients (e.g., telephone, zoom) changed the nature of the engagement with service users, the convenience of virtual communication during COVID-19 public health restrictions may have led to increased responses to keyworker contacts and higher levels of engagement with interventions compared to periods without public health restrictions.

The positive relationship between the number of keyworker contacts and engagement with psychosocial interventions (e.g., CBTp, BFT and IPS) highlights the importance of keyworking positions. These findings indicate that keyworker contacts were highly effective in facilitating engagement with additional EIP services after adjusting for socio-demographic, substance use and clinical factors. The ENDEARVOR trial in the UK found that EIP keyworker attitudes to IPS was a mediating factor influencing IPS uptake [29]. It is not known within the current study exactly what their own attitudes were to specific pillar interventions but the keyworkers were central to coordinating engagement with these services. The high rates of service user engagement with these interventions would suggest that keyworkers were positively disposed to interventions and actively promoting them. Given the strong effects of keyworker engagement, EIP services should ensure that there is a sufficient complement of keyworker staff in place to support frequent contact with service users to facilitate engagement with other EIP services including CBTp, BFT and IPS. These strong positive effects were observed in spite of challenges with staffing capacity and staff maternity leave and COVID-19 public health measures during the study period.

However, this does require careful monitoring and resourcing so that caseloads, for example, remain within the identified parameters of the MoC (e.g., each keyworker was to have no more than 15 cases at any one time although this was exceeded in Site A and Site B during the process evaluation). Caseload management requires caseload analyses, scheduling of care, delegation, prioritisation workforce planning and teamwork. At the centre of this is the principle of the National Clinical Care Programmes which is to ensure quality, effectiveness, efficiency and cost effectiveness. To achieve this also requires accurate data on referrals, activity, ongoing caseload size. Linked to this is the need to understand the average anticipated duration of treatment for service users and accurate discharge processes. Currently, in the Irish context there is no method within the health service which looks to support accurate caseload analyses and planning.

### Policy, practice, research implications

These findings contribute to existing evidence demonstrating that the keyworking position in EIP services plays a critical role in realising the benefits of these interventions. The EIP Keyworker role is new in Ireland. The findings form this research are supporting a formalisation of this new role via the development of a dedicated EIP Keyworker Job Grade in the Irish Health Service. A previous review of studies from ten countries identified three themes of outcomes that are mediated by keyworker engagement: decreased psychosis severity; lifestyle improvements and reduced organisational barriers [10, 30]. This study advances the understanding of keyworker effects through qualitative evidence of keyworkers functioning as a “linchpin” to the service, while the positive response association between keyworker contacts and engagement with other services provides quantitative support for keyworkers reducing the organisational or structural barriers to service access [10]. Existing studies have also found that the aspects of EIP services that were most valued by service users were therapeutic relationship and opportunities for agency that created a personalised recovery journey [13, 15]. Since the keyworker is the principal contact for service users, functions to coordinate care and reduces barriers to other services, health systems and policies adopting EIP services need to recognise the importance of these positions in maximising service impact. Given the demands of these roles and how integral the keyworker role is to facilitating engagement, it is critical that these positions be filled by suitably experienced and trained staff and that caseload numbers are protected.

Key to effective EIP delivery is building relationships with service users [15]. This often takes time to establish and build trust between the service users and individual staff. EIP policy within Ireland states that a keyworker should have no more than 15 service users on their caseloads at any one time [6]. This is in recognition of the intensity of the work that needs to occur to properly support service users. Linked to this is also the close collaboration between staff in relation to the building and progressing of a treatment plan. This important work which is a building block of EIP can be hard to capture and quantify. Despite the resourcing pressure on all healthcare systems, the findings of this study underlie the need for EIP keyworker posts to be recruited and resourced in line with evidence-based practice.

Athough the data capture system used for the present study was resource and time intensive, the availability of prospective service data provided critical information to inform clinical practice, as well as policy. This data can indicate service strengths and deficiencies to better inform health service spending and efficiency. Novel models of EIP service design (e.g., hub and spoke) can also be evaluated relative to other approaches to inform service design in unique geographic and demographic settings. Using data capture methods such as electronic medical records could also assist with future research investigating long term service users’ trajectories (e.g., relapse, hospitalisation) as well as personalised care programs for distinct clinical profiles (e.g., multimorbidity, substance use).

This research comprises a mixed methodological approach, presenting both quantitative and qualitative data and reflecting the real-world implementation of a new service. Much of the data on EIP comes from randomised controlled trials, which while of great methodological value, may have less ecological validity. The prospective data collection commenced soon after the service was implemented and provided longitudinal data on service engagement throughout the life-cycle of implementation. This research was conducted by individual evaluators, who are not EIP clinicians, reducing the likelihood of observational bias. Finally, data from three clinical sites is described, increasing the study’s representativeness.

This study does not include any comparative data from sites where EIP was not implemented, or where a different form of care was trialled. This was beyond the feasibility and aims of this process evaluation study however. Secondly, data on service users were inputted by staff members and this may have introduced some unconscious bias where staff hoped to present a positive view of this newly fledging service. Finally, service users were proposed for qualitative interviews by EIP staff, so there is a possibility that those who did not wish to be interviewed or who were not asked by staff, may have had more negative experiences of EIP, not reflected in this data.

## Conclusion

Together, these findings demonstrate the integral role of keyworkers in coordinating care and facilitating engagement with EIP services. This perspective was reflected in qualitative interviews from service users as well as EIP staff and clinicians. Quantitative data showed that monthly keyworker engagement increased the odds of CBTp, BFT and IPS engagement by three to five fold, further supporting the importance of the keyworker. Given the importance of these positions, health systems should ensure that EIP programmes identify qualified and experienced staff to fill these roles, as well as allocate the appropriate funding and protected time to support keyworker engagement and impact.

## Data Availability

The availability of quantitative and qualitative data, for meta-analyses or narrative synthesis, is available on reasonable request by contacting the corresponding author.
